# Death of One of Pasteur’s Patients

**Published:** 1886-05

**Authors:** 


### DEATH OF ONE OF PASTUERS PATIENTS.

An autopsy was made by Drs. Cornil and Roux on the body of the Russian patient of Pasteur, who died of rabies in the Hotel Dieu. The bites on the face were very extensive in two places, the upper lip was pierced through and torn aside, leaving the gum exposed as far as the first molars. Another wound in the temporal region, just above the zygoma, was also very deep, and in it was found a fragment of a canine tooth of the wolf. The brain, medulla and spinal cord presented no abnormal appearance.—*Progres Medical.*

